# A multi-scale feature extraction fusion model for human activity recognition

**DOI:** 10.1038/s41598-022-24887-y

**Published:** 2022-11-30

**Authors:** Chuanlin Zhang, Kai Cao, Limeng Lu, Tao Deng

**Affiliations:** 1 School of Mathematics and Computer Science, Northwest Minzu University, Lanzhou, 730030 People’s Republic of China; 2 Key Laboratory of China’s Ethnic Languages and Information Technology of Ministry of Education, Northwest Minzu University, Lanzhou, 730030 People’s Republic of China; 3 Key Laboratory of Streaming Data Computing Technologies and Application, Northwest Minzu University, Lanzhou, 730030 People’s Republic of China

**Keywords:** Computational science, Computer science, Information technology

## Abstract

Human Activity Recognition (HAR) is an important research area in human–computer interaction and pervasive computing. In recent years, many deep learning (DL) methods have been widely used for HAR, and due to their powerful automatic feature extraction capabilities, they achieve better recognition performance than traditional methods and are applicable to more general scenarios. However, the problem is that DL methods increase the computational cost of the system and take up more system resources while achieving higher recognition accuracy, which is more challenging for its operation in small memory terminal devices such as smartphones. So, we need to reduce the model size as much as possible while taking into account the recognition accuracy. To address this problem, we propose a multi-scale feature extraction fusion model combining Convolutional Neural Network (CNN) and Gated Recurrent Unit (GRU). The model uses different convolutional kernel sizes combined with GRU to accomplish the automatic extraction of different local features and long-term dependencies of the original data to obtain a richer feature representation. In addition, the proposed model uses separable convolution instead of classical convolution to meet the requirement of reducing model parameters while improving recognition accuracy. The accuracy of the proposed model is 97.18%, 96.71%, and 96.28% on the WISDM, UCI-HAR, and PAMAP2 datasets respectively. The experimental results show that the proposed model not only obtains higher recognition accuracy but also costs lower computational resources compared with other methods.

## Introduction

In recent years, human activity recognition has attracted great interest from an increasing number of researchers due to its wide applications in everyday life such as healthcare^1^, motion analysis^2^, intelligent monitoring system^3^, and smart home^4^. HAR focuses on analyzing the acquired human behavior information to understand and predict specific human behavior. The information is obtained in various ways, including accelerometer, infrared, RFID, and video. Different data patterns represent different ways of encoding human behavior, providing different values and sources of information. Currently, HAR can be broadly classified into two categories based on the source of data acquisition: video-based^5–7^ and sensor-based^8–10^. Video-based systems mainly use devices such as cameras to capture videos and images to recognize daily life activities and human behaviors through techniques in computer vision. Despite some good performance they show, they are susceptible to environmental factors such as lighting conditions and target occlusion, and they also exist privacy issues^11^. In contrast, sensor-based systems use environmental or wearable sensors to identify human activity. Sensors are widely embedded in smart devices, such as smartphones and smartwatches. The ubiquity and indispensability of smart devices in our daily lives, coupled with their portability and computational power, have made sensor-based systems a major approach to HAR research.

Recognition accuracy is a constant goal, and over the past few decades, researchers have adopted many traditional machine learning (ML) methods, including support vector machine (SVM)^12^, random forest^13^, and k-nearest neighbors (kNN)^14^, to recognize different human activities using data obtained from smartphones or wearable sensors. The accuracy of these methods depends greatly on the quality of the extracted features. This requires researchers to not only have some prior knowledge to manually design the extracted features according to the application scenario, such as time^15^, frequency^16^, and time–frequency domain features, but also feature selection and dimensionality reduction to select the representative features. Besides, the extracted features are always restricted to the specific scene and fail to be used in other similar environments^17^.

DL methods have been very mature and successful in computer vision (CV)^18,19^, target detection^20,21^, and natural language processing^22^. Now, DL is also being used more and more widely in HAR, because it could automatically extract high-level features from the original data, effectively avoiding the complex and time-consuming manual feature extraction process, and could be applied to any case of HAR. Several DL methods, including CNN^23^, Recurrent Neural Networks (RNN)^24^, and Extreme Learning Machine (ELM), have been widely used to learn feature representations of sensor data and achieve better recognition results.

RNN were originally designed to process serial data, and the data collected by various types of sensors are basically one-dimensional time series^25^, so it is well suited for the study of sensor-based HAR. As the original RNN suffers from the gradient disappearance/explosion, GRU^26^ and long short-term memory (LSTM) were introduced to solve this problem, and these two naturally become the mainstream^27^. Although RNN improves the accuracy of activity recognition, its unique mechanism that the computation of the later step depends on the result of the previous step and the computation order can only be performed sequentially leads to problems such as high number of model parameters and long training time compared to other methods, which is a challenge for terminal devices with limited memory and computational power like smartphones and smartwatches. Compared with RNN, CNN has local connectivity and weight-sharing mechanisms, which make the model have fewer parameters and faster training, thus a large number of studies on sensor-based activity recognition based on CNN have also emerged^28^. The feature extraction capability and recognition accuracy of CNN depend on the depth and width of the network. However, with the network getting wider and deeper, the feature extraction capability and recognition accuracy increase. This inevitably leads to an increase in the model parameters dramatically. Therefore, whatever method is chosen, we need to consider both the accuracy and the model size.

In this study, we propose a multi-scale feature extraction fusion model with different convolutional kernel sizes. Firstly, the proposed model uses different convolutional channels, of which each has a different convolutional kernel size. Secondly, a separable convolution^29^ different from the classical convolution is used to reduce the model parameters and ensure its accuracy, and then each channel is connected with a GRU after the convolution operation to extract the local features and long-term dependencies of the original data, further enhancing the feature extraction capability of the network. Finally, the features extracted from each channel are concatenated as the final feature representation.

The main innovations and contributions of this work are:

1. The proposed model combines the functions of CNN and GRU to achieve the multi-dimensional automatic extraction of the spatio-temporal features of the original data.
2. Multiple feature extraction channels are used, and each channel uses convolution kernels of different sizes, which can realize the extraction of features of different scales and improve the richness of feature extraction.
3. The structure of the model is carefully designed, including the content and parameters of each layer, and the separable convolution is used to replace the classical convolution, improving the accuracy of model identification and reducing the model parameters.

The rest of this study is organized as follows. Section “Related work” presents related work on sensor-based HAR using traditional ML methods and DL methods. Section “Material and methods” describes the datasets used in this study and the main stages of the HAR architecture, including data preprocessing, feature extraction, and the proposed model. The experiments and results are illustrated and discussed in Section “Experimental results”. Finally, Section “Conclusion” presents the conclusions drawn.

## Related work

Traditional ML algorithms require domain expertise and tedious feature engineering to obtain feature representation of the raw sensor data and identify activities by classifiers such as decision trees^30^ or Naive Bayes^31^. For example, Lee et al.^32^ collected angular velocity data through a gyroscope attached to the foot and used a decision tree model to classify behaviors such as walking, running, upstairs, and downstairs. Ignatov et al.^33^ proposed an online time series segmentation method and achieved the classification of six behaviors with 94% accuracy using principal component analysis and KNN. Fleury et al.^34^ used accelerometers, magnetometers, and infrared sensors to collect data, conducted the principal component analysis on the extracted features to obtain 10 main features, and achieved the recognition of 35 behaviors by training called multi-SVM model with 86% accuracy.

In contrast, DL algorithms, such as CNN and RNN, do not require special consideration of specific settings, and automatically perform feature extraction and classification, with good results in various sensor-based HAR scenes^35^. Ignatov^36^ proposed a network architecture that combines local features extracted by CNNs with statistical features to capture the global features of sensory data and pass the feature set to the fully connected layer for classification. Zhang et al.^37^ mapped the motion sensor data captured by the wearable sensor into the single-pixel column, multi-channel images, which were then fed into a U-Net network to complete the pixel-level activity recognition function. In Ref.^38^, the authors designed a bidirectional LSTM model for HAR using time series data from the UCI-HAR dataset. To extract robust features from raw sensor data automatically and efficiently, Ronao and Cho^39^ proposed a model consisting of alternating convolutional and pooling layers. The extracted features are then passed to the fully connected layers and softmax layers to predict human activity. Murad et al.^40^ proposed the use of deep recurrent neural networks (DRNNs) for building recognition models that are capable of capturing long-range dependencies in variable-length input sequences and compared them with methods such as KNN and SVM, and experiments showed that DRNNs can achieve better recognition results. Ronald et al.^41^ proposed the iSPLInception, a DL model motivated by the Inception-ResNet architecture from Google, that not only achieves high predictive accuracy but also uses fewer device resources. Lohit et al.^42^ paid more attention to the data processing stage and proposed a temporal transformer network (TTN) for the possible temporal dislocation in human activity analysis.

Another design paradigm, which is very popular now is that researchers have been combining the strengths of different networks to develop hybrid models. Ordonez and Roggen^43^ combined deep CNN and LSTM for the classification of 27 gestures and 5 actions. Xu et al.^44^ also introduced a multichannel structure, consisting of Google's Inception network and LSTM in HAR to automatically extract richer features. Xia et al.^45^ proposed an LSTM-CNN model, made up of two LSTM layers, a convolutional layer, a global average pooling (GAP) layer, a batch normalization (BN) layer, and a softmax layer, and evaluated the model on three publicly available datasets. Karim et al.^46^ proposed transforming the existing univariate time series classification models, the Long Short Term Memory Fully Convolutional Network (LSTM-FCN) and Attention LSTM-FCN (ALSTM-FCN), into a multivariate time series classification model by augmenting the fully convolutional block with a squeeze-and-excitation block to further improve accuracy.

Despite the good results DL methods have achieved in HAR, issues such as the extraction of richer and more effective features and balancing computational cost with recognition accuracy remain some of the major challenges in the HAR field today. This study proposes a fusion model capable of extracting features of different scales and types. Meanwhile, a separable convolution is used in the proposed model to minimize the model size.

## Material and methods

### Datasets

In this work, we adopt three publicly available datasets: WISDM, UCI-HAR, and PAMAP2 datasets. The WISDM dataset is a single-sensor unbalanced dataset of a relatively larger number of samples. The UCI-HAR dataset has the same behavior category as the WISDM, but in uniform distribution of samples for behavior categories. The PAMAP2 dataset has the most activity categories among the three datasets. The basic information of the three datasets is shown in Table 1, and their details are as follows.

**Table 1:** Essential information of used datasets.

Datasets	Volunteers	Sensors	Sampling rate (Hz)	Activities	Samples
WISDM	36	Accelerometer	20	6	1,098,207
UCI-HAR	30	Accelerometer, Gyroscope	50	6	748,406
PAMAP2	9	Accelerometer, Gyroscope, heart rate monitor	100	18	2,872,533

#### WISDM dataset^24^

The WISDM dataset is a benchmark HAR dataset derived from the Wireless Sensor Data Mining Laboratory and contains a total of 1,098,207 samples. This is an activity recognition dataset collected for 36 users who perform daily activities, including the six behaviors of walking, sitting, jogging, downstairs, upstairs, and standing. These data were obtained by the experimental users with an Android phone in their front leg pocket, using the phone's built-in accelerometer sensor with a sampling frequency of 20 Hz.

#### UCI-HAR dataset^11^

This data set was prepared by 30 volunteers between the ages of 19 and 48 years old via a waist-mounted (Samsung Galaxy SII) smartphone. Each person performs six activities (walking, upstairs, downstairs, sitting, standing, and laying) and the data is collected at a constant rate of 50 Hz using the smartphone's built-in gyroscope and accelerometer. The raw data contained nine features coming from three-axis body acceleration, three-axis total acceleration, and three-axis angular velocity. These experiments were videotaped to manually label the data.

#### PAMAP2 dataset^47^

The PAMAP2 collects various activities from 9 volunteers (1 female, 8 males), including 12 protocol activities (lying, sitting, standing, walking, running, cycling, nordic walking, ironing, vacuum cleaning, rope jumping, ascending and descending stairs) and 6 optional activities (watching TV, computer work, car driving, folding laundry, house cleaning, playing soccer). The activity data were recorded by IMUs (inertial measurement units) sensors installed at different positions of the human body (hand, chest, and ankle). A total of 52 features were captured at a sampling rate of 100 Hz.

### Proposed HAR system

**Figure 1:**
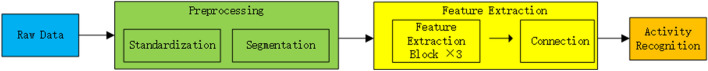
Architecture of the proposed HAR system.

**Figure 2:**
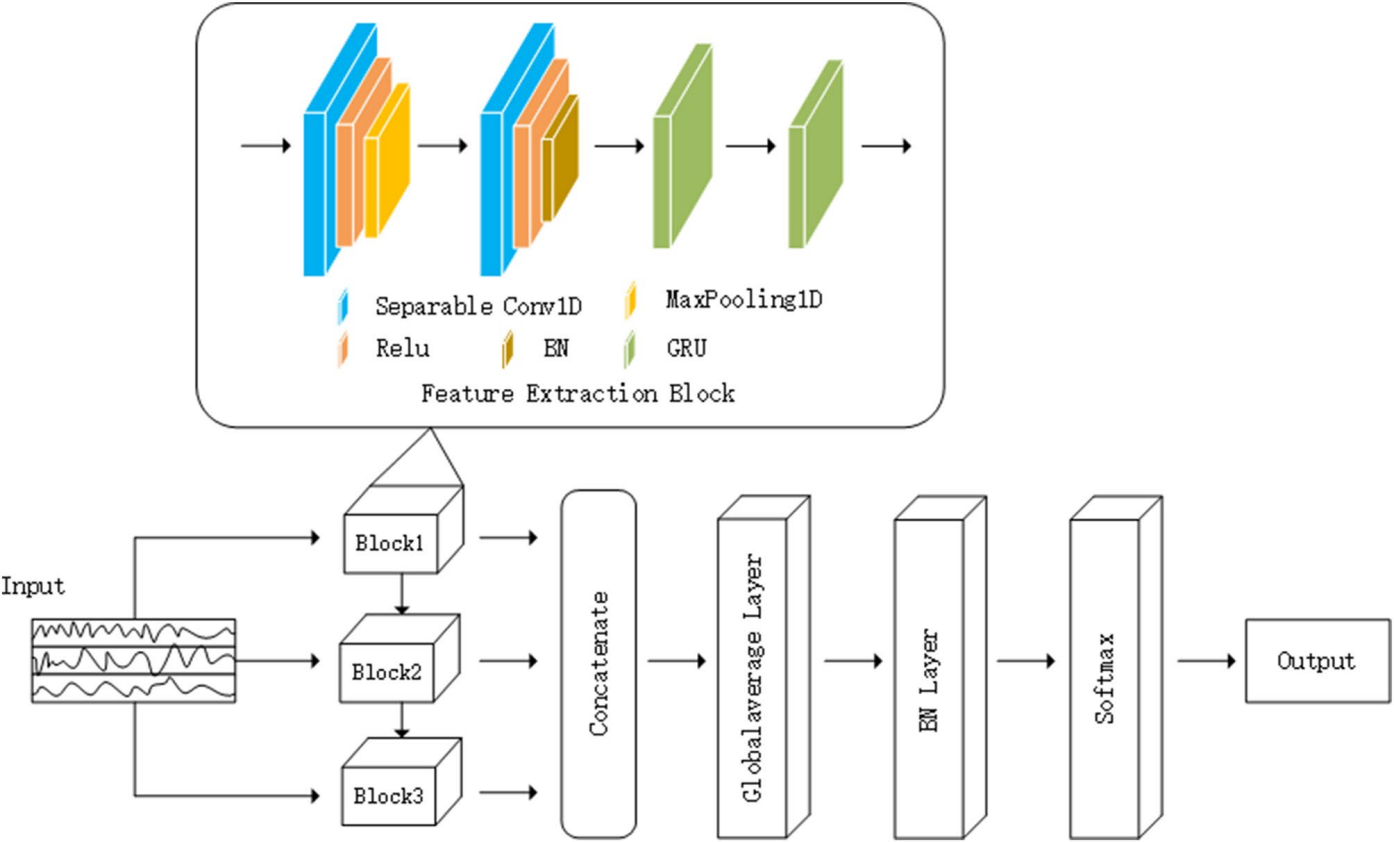
The network architecture of the proposed model.

In this section, we have discussed the main phases of the proposed HAR system as illustrated in Fig. 1. The detailed description of the proposed network architecture is shown in Fig. 2. Firstly, the acquired raw data, which can not be fed into the HAR model directly, needs to go through a series of preprocessing processes such as data cleaning, standardization, and data segmentation, to transform into the data acceptable to the network. Then the feature extraction network captures the effective feature representation of the data, and finally, the model recognizes and outputs the classification results of human behavior.

#### Data pre-processing

First of all, abnormal data such as noise and missing values will inevitably be generated during data acquisition due to the complex environment or unstable sampling rate. Filtering noise and linear interpolation are used to process these data, which helps the accuracy of model recognition. The data are then normalized (all values have a mean zero and standard deviation of one) and the normalized data are passed to the segmentation stage, which is a very important step in preparing the sensor data for the HAR model. For its simplicity and high computational efficiency, the sliding window method based on fixed length is used for data segmentation. The length of the sliding window is set to 128 with an overlap rate of 50% for the datasets of WISDM, UCI-HAR, and PAMAP2.

#### Feature extraction

Feature extraction is the key step in the process of HAR and crucial to the subsequent classification result. A multi-scale feature extraction method is presented to extract the rich and deep level of features of the data. The method consists of three main feature extraction blocks, which have a similar structure and differ only in the size of the convolution kernel. The features extracted from each feature extraction block at different scales join together as the final feature representation.

**Figure 3:**
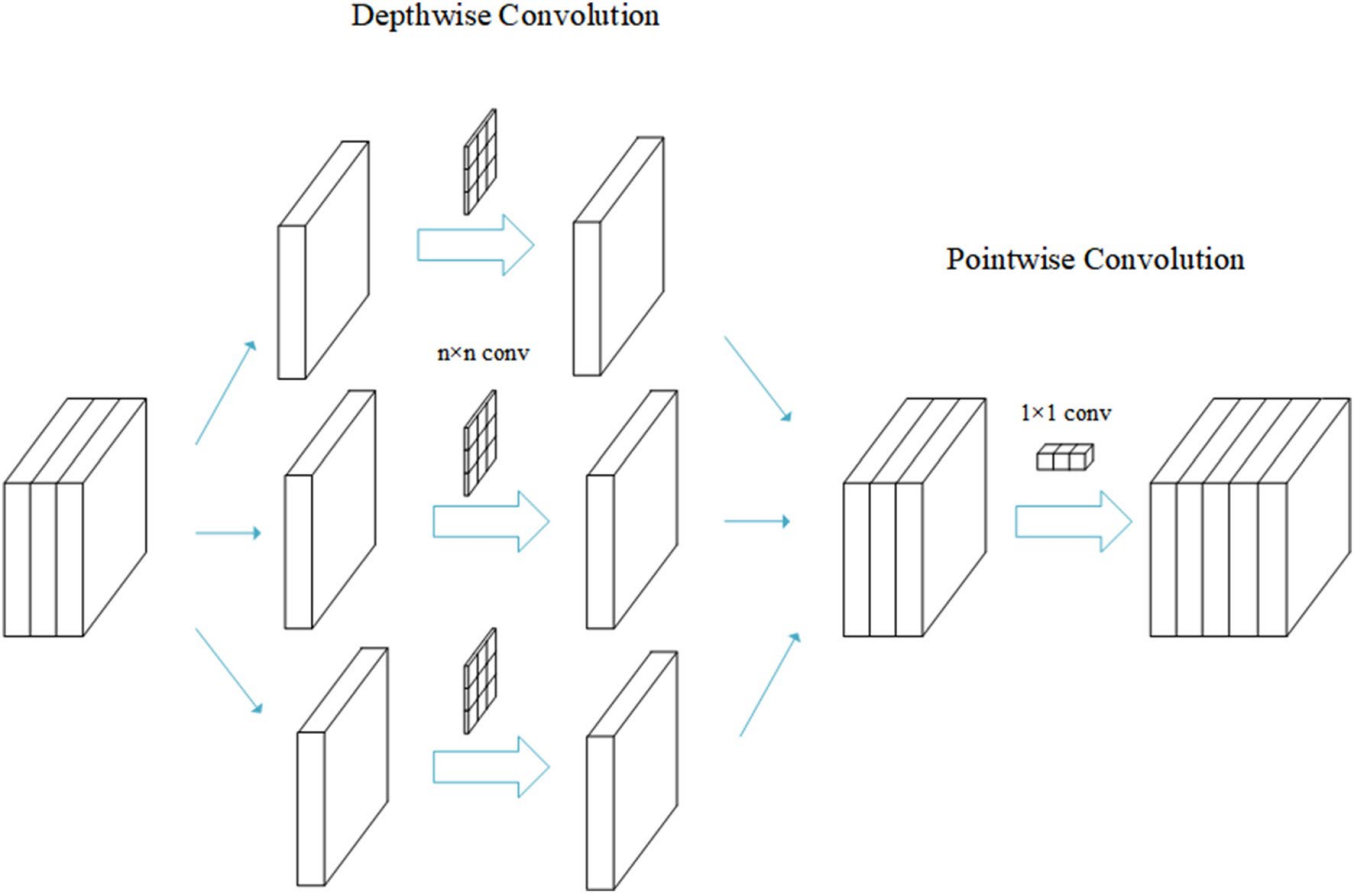
Separable convolution schematic.

The feature extraction block includes two convolutional layers, one pooling layer, one BN layer, and two GRU layers, which combines the advantages of CNN and GRU to complete the automatic extraction of spatio-temporal features of the original data, and its detailed structure is shown in the box in Fig. 2. As can be seen from the Fig. 2, the separable convolution instead of the classical convolution is used, because it can guarantee the recognition accuracy while reducing the computational cost. This meets the requirements of HAR for minimizing the number of parameters and the size of the model. The separable convolution, though has the same output dimension as classical convolution, differs greatly in the implementation process. It consists of two main stages: depthwise convolution and pointwise convolution, as shown in Fig. 3. In the first stage, one convolutional kernel responds to one channel, and the number of convolutional kernels is the same as the number of channels in the previous layer, thus the number of completed feature maps is the same as the number of channels in the input layer. This operation only performs the convolution operation for each channel independently, but fails to effectively utilize the feature information of different channels at the same spatial location. Therefore, the pointwise convolution stage is needed to make full use of the position information of different channels. The operations in the pointwise convolution stage are very similar to the classical convolution operations, with a convolution kernel of size 1 $\times$ 1 $\times$ M (M is the number of channels in the previous layer). The convolution operation here will weigh the feature maps obtained in the previous stage in the depth direction to generate new feature maps.

Human activity data collected through wearable sensors and smartphones are time series data, which means that adjacent variables are strongly correlated in time. CNN is able to capture short variations in time series signals by convolutional kernels, which considers each part of the sensor data as independent and extracts features for these independent parts of the data without considering the temporal contextual information between the parts of the data. Thus, CNN is good at dealing with extracting short-term and local features. However, the data of HAR are long time series data, and it is essential to consider the temporal context between the parts of the data for identifying the activities more accurately. Conventional RNN are inefficient in capturing long-term dependencies because the gradient may disappear or explode when it comes up with long sequence data. GRU, a variant of RNN, is well suited for processing long-time sequence data, and it can effectively address the problems of gradient disappearance and short-term memory existing in Conventional RNN. GRU integrates gating units into the conventional cyclic unit, enabling it to remember much earlier information and predict the current state based on information obtained from previous states. These gates help GRU to determine when and how much information from the past is sent to future states, and therefore, GRU is good at capturing long-term dependencies in time series data. As mentioned above, the combination of CNN and GRU has great advantages to extract local features and long-term dependencies of the data. The range of local feature extraction is controlled by adjusting the size of the convolutional kernels and affects the extraction of long-term temporal features. In the end, the channels with different sizes of convolutional kernels are connected to form the proposed model.

#### Proposed model

The proposed model has three channels with different filter sizes, and each channel receives three-dimensional data including samples, timestamps, and channels, where samples represent the number of windows in the used dataset, timestamps represent the size of the sliding window, and channels represent the number of input features. The number of filters in the two convolutional layers of each channel are 64 and 128, and the filter sizes of different channels are 3, 5, and 7, respectively. A max pooling layer with a pool size of 2 exists between the two convolutional layers. Then a BN layer is connected to speed up the training of the network and control overfitting. The feature output by the BN layer is then fed to two GRU layers, each of which contains 128 units. The features captured by the three channels are concatenated and sent to the GAP layer to reduce the model parameters, and finally the softmax layer for recognition output.

## Experimental results

### Experimental setup

The data set should be reasonably partitioned to evaluate the proposed model. If the variability of users is ignored and the entire data are randomly divided into training and test sets, the classification model may see the same person’s activity in both the training and test sets. This may gain higher accuracy, but does not properly reflect the true performance of the model. Therefore, the data set should be partitioned by the user-id to make the model fit real-life situations. In the WISDM, the first 30 users’ data are selected as the training set, and the following 6 users’ data as the test set; In the UCI-HAR, the activity data of 21 volunteers are selected for training and the activity data of the other 9 volunteers are selected for testing; In the PAMAP2, the data collected from the sixth and seventh subjects are used for testing, while the data from the other seven subjects for the training set. The details of the three datasets are shown in Table 2.

**Table 2:** Instances of three public datasets.

	WISDM	UCI-HAR	PAMAP2
Training set	14,035	7352	19,700
Test set	3121	2947	6727

The proposed model is implemented on the Keras API of the TensorFlow backend. Adam optimizer is adopted for the training of the proposed model with a learning rate of 0.001. To measure the loss of the proposed classification model, categorical cross-entropy is used. In all experiments, the model was trained on NVIDIA GeForce RTX 3060 GPU with batch sizes of 128 for 100 epochs. Table 3 shows the hyperparameters selected for the experiments, other parameters all use default values.

**Table 3:** List of hyperparameters used in this work.

Period	Hyperparameters		Values
Data preprocessing	Window size		128
	Step		64
Feature extraction	Separable convolution_1	Kernel size	3/5/7
		Filters	64
	Maxpooling	Pooling size	2
	Separable convolution_2	Kernel size	3/5/7
		Filters	128
	GRU_1 neurons		128
	GRU_2 neurons		128
Training	Optimizer		Adam
	Number of epochs		100
	Batch size		128
	Learning rate		0.001

### Performance metrics

Four evaluation metrics of accuracy, precision, recall, and F1-score are used to evaluate the performance of the proposed model. These metrics are mathematically expressed as:

1 $$Accuracy = \frac{TP + TN}{{(TP + FP + FN + TN)}}$$

2 $$Precision = \frac{TP}{{(TP + FP)}}$$

3 $$Recall = \frac{TP}{{(TP + FN)}}$$

4 $$F1 - score = \frac{2 \times Precision \times Recall }{{ Precision + Recall }}$$

where TP = True Positives, FN = False Negatives, TN = True Negatives, and FP = False Positives. In addition to the above four evaluation indexes, a confusion matrix (CM) is also used to show the specific classification results of each category. The CM provides a clear understanding of the classification of unbalanced data and also can calculate the classification accuracy of each category quantitatively.

### Results

#### WISDM dataset results

Table 4 shows the experimental outcome of the proposed model and the model using the classical convolution on the WISDM dataset. Both models have the same structure, and the only difference is that one uses the separable convolution, while the other adopts the classical convolution. The accuracy of our method reached 0.9718 whereas the precision, recall, and F1-score are 0.9726, 0.9718, and 0.9717 respectively. As can be seen in Table 4, our method is not only better than the method using the classical convolution in recognition accuracy but also reduces the number of parameters by 13.8%. It shows that the separable convolution is superior to the classical convolution in processing one-dimensional time series signals.

**Table 4:** Performance comparison of the proposed model with the model using classical convolutions on the WISDM dataset.

Methods	Accuracy (%)	Precision (%)	Recall (%)	F1-score (%)	Parameters
The model using the classical convolution	96.32	96.47	96.32	96.34	723,590
Proposed model	97.18	97.26	97.18	97.17	623,987

Table 5 compares the accuracy and F1 score of the proposed model with existing models on the WISDM. Reference^36^ combined features automatically extracted by CNN with statistical features extracted manually to obtain richer features, while Ref.^37^ adopted the U-NET model more commonly used in the CV for the HAR system. Both^43^ and^45^ used a fusion model combining the advantages of feature extraction of CNN and LSTM, which mainly differ in the connection sequence of CNN and LSTM in network structure. As shown in Table 5, the proposed models show higher performance compared to the baseline experiments, as it not only extracts local features and long-term dependencies from the original data but also limits the range of extracted features by combining different convolutional kernel sizes. Thus, the proposed model could obtain more effective features and higher recognition accuracy.

**Table 5:** Performance comparison of the proposed model with various DL-based models on the WISDM dataset.

Models	Accuracy (%)	F1-score (%)
LSTM^24^	95.78	95.73
DeepConvLSTM^43^	–	93.01
LSTM + CNN^45^	–	95.85
CNN + Statistical features^36^	93.3	–
U-Net^37^	96.4	96.5
Proposed model	97.18	97.17

**Figure 4:**
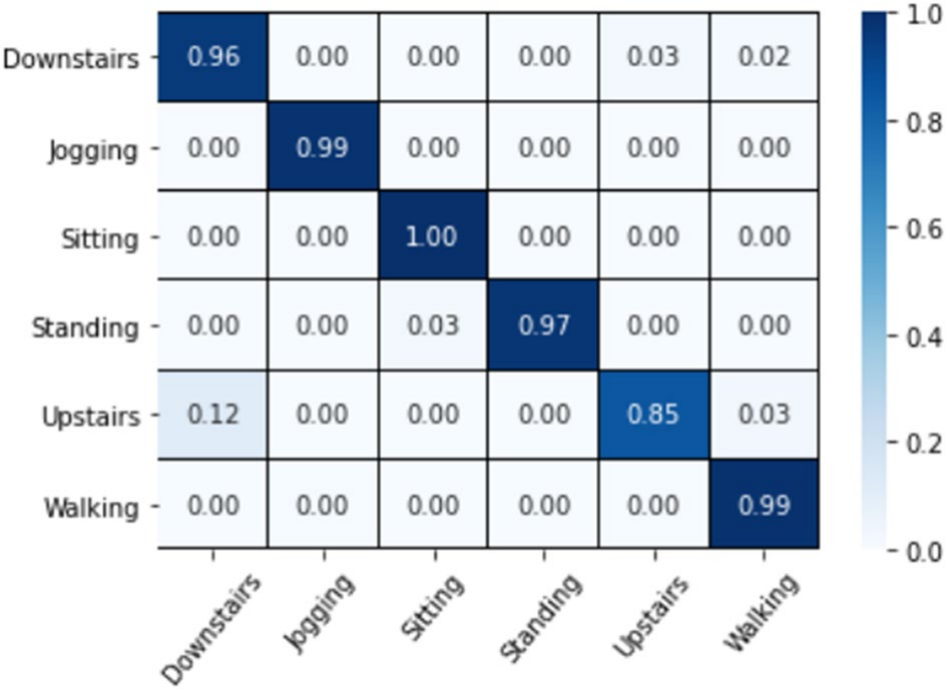
Confusion matrices for the proposed model on the WISDM dataset.

The CM, shown in Fig. 4, is obtained by evaluating the trained proposed model on the test set. It can be found that static actions such as sitting and standing, and dynamic actions of jogging and walking have higher recognition rates. Compared with other movements, the misclassification rate of dynamic movements of the upstairs and the downstairs is higher. Nearly 12% of the upstairs is misclassified as the downstairs, while 3% of the downstairs is misclassified as the upstairs, this is because the upstairs is more similar to the downstairs in movement range.

#### UCI-HAR dataset results

Table 6 shows the classification results of the proposed model and the model using the classical convolution on the UCI-HAR dataset. Our method achieves an accuracy of 0.9671, precision of 0.9683, recall of 0.9671, and F1-score of 0.9672 respectively on the test set. It also outperforms the model using the classical convolution in terms of recognition accuracy and model size.

**Table 6:** Performance comparison of the proposed model with the model using classical convolutions on the UCI-HAR dataset.

Methods	Accuracy (%)	Precision (%)	Recall (%)	F1-score (%)	Parameters
The model using the classical convolution	96.03	96.10	96.03	96.03	729,350
Proposed model	96.71	96.83	96.71	96.72	625,229

The performance comparison of our model with other models is presented in Table 7. As shown in Table 7, our proposed model performs much better than InnoHAR and iSPLInception, both of which are branching structures, and it is the same as that of MLSTM-FCN, a hybrid model with a squeeze-and-excitation block that has the best performance in the comparison model. It can be illustrated that our method can extract more distinguishing features and obtain better recognition results in both cases of single-sensor and multi-sensor.

**Table 7:** Performance comparison of the proposed model with various DL-based models on the UCI-HAR dataset.

Models	Accuracy (%)	F1-score (%)
CNN^28^	92.71	92.93
Stacked-LSTM^27^	93.13	–
Res-BiLSTM^38^	93.6	93.5
InnoHAR^44^	–	94.5
LSTM + CNN^45^	–	95.78
MLSTM-FCN^46^	96.71	–
iSPLInception^41^	95.09	95
Proposed model	96.71	96.72

**Figure 5:**
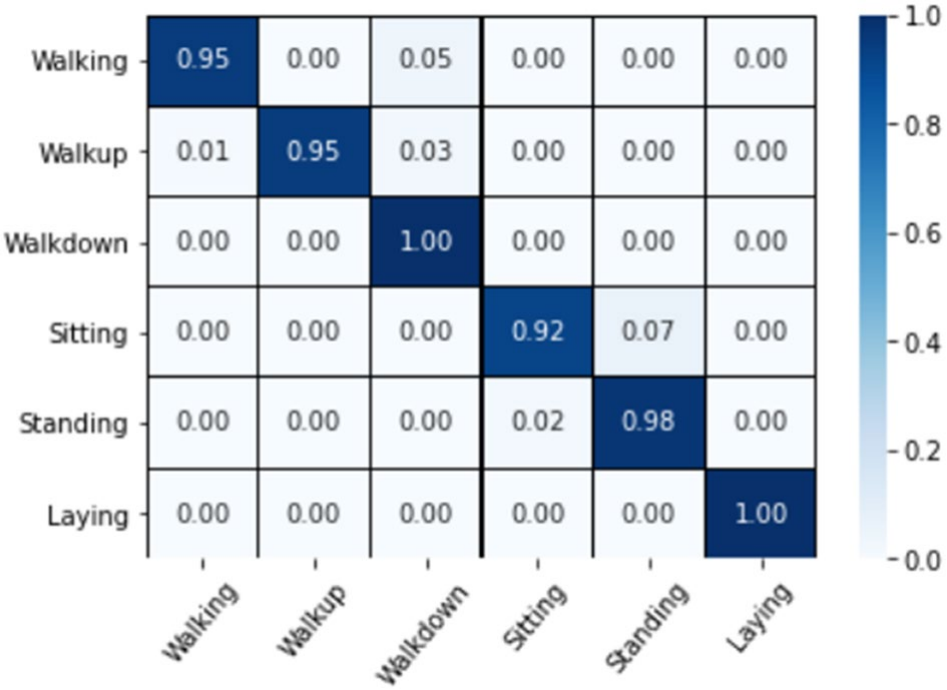
Confusion matrices for the proposed model on the UCI-HAR dataset.

The CM is able to describe the classification results of different categories in detail, and the CM for this dataset is displayed in Fig. 5. The samples of different behavioral categories in this dataset are relatively balanced, and the sitting activities had the highest misclassification rate among all behavioral activities, with 7% being incorrectly identified as the standing, while 2% of the standing activities were predicted as the sitting, this is probably because the sitting is very similar to the standing.

#### PAMAP2 dataset results

In this dataset, 11 protocol activities are selected for experiments except for rope jumping. That is because the rope jumping in protocol activities has very little recording time, and even some users did not perform this activity. The other activities are more balanced categories. The optional activities are not selected due to that only a small number of users perform these activities. Table 8 shows the experimental results of the proposed model and the model using the classical convolution on the PAMAP2 dataset. Table 9 shows the comparison of the recognition performance of the proposed model and other models on PAMAP2, and it can be seen that the proposed model has a better recognition effect than the benchmark model.

**Table 8:** Performance comparison of the proposed model with the model using classical convolutions on the PAMAP2 dataset.

Methods	Accuracy (%)	Precision (%)	Recall (%)	F1-score (%)	Parameters
The model using the classical convolution	95.76	95.92	95.76	95.71	771,275
Proposed model	96.28	96.37	96.28	96.27	634,775

**Table 9:** Performance comparison of the proposed model with various DL-based models on the PAMAP2 dataset.

Models	Accuracy (%)	F1-score (%)
BiLSTM^28^	89.52	89.40
CNN^28^	91.00	91.16
COND-CNN^48^	–	94.01
Proposed model	96.28	96.27

**Figure 6:**
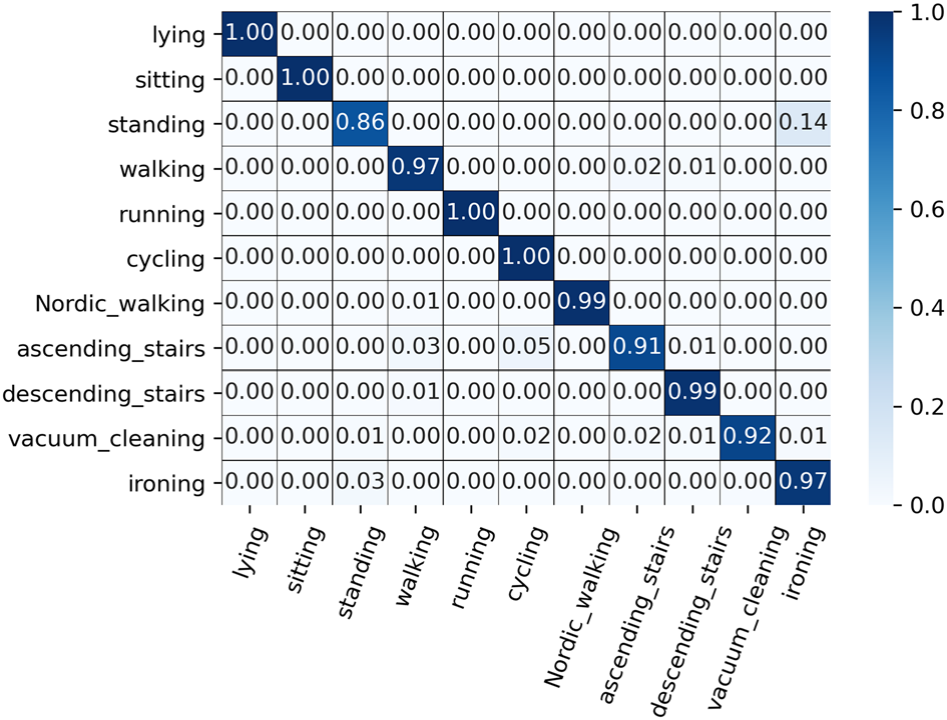
Confusion matrices for the proposed model on the PAMAP2 dataset**.**

The CM is shown in detail in Fig. 6. It can be seen from Fig. 6 that the behaviors of lying, sitting, running, and cycling are completely recognized correctly, while standing has the lowest recognition accuracy, and some samples are wrongly classified as ironing in which people are standing while their hands are performing actions.

#### Performance comparison of the models using different filter sizes

A multichannel structure combing convolutional kernels of different sizes with GRU is used to extract much richer features from the raw data during the training process, thus obtaining higher recognition accuracy. To explore the impact of multiple filter sizes used in the proposed model on classification performance, we compare the proposed model with the other three models with different single-size filters, shown in the small box in Fig. 2, and with the same multichannel model with different filter sizes. The three single-size filter models differ only in filter size, and the three filter sizes are 3, 5, and 7, respectively. The same multichannel model has the same model structure as the proposed model in Fig. 2, except that the convolution kernel sizes of the three channels are changed to 3, 7, and 11. The comparison results are shown in Table 10. From Table 10, it is observed that the proposed model outperforms the models using single-size filters and multichannel models with different filter sizes, showing its effectiveness in the combination of different filters size and its rationality of convolution kernel size setting.

**Table 10:** Performance comparison of the proposed model with different filter sizes.

Models	WISDM		UCI-HAR		PAMAP2	
	Accuracy (%)	F1-score (%)	Accuracy (%)	F1-score (%)	Accuracy (%)	F1-score (%)
Single-size filter model (filter size = 3)	93.34	93.53	94.74	94.68	94.20	94.18
Single-size filter model (filter size = 5)	94.55	94.63	95.08	95.05	94.80	94.76
Single-size filter model (filter size = 7)	94.78	94.78	95.32	95.33	95.05	95.00
Multichannel model (filter sizes = 3, 7, 11)	95.77	95.81	95.93	95.92	95.24	95.18
Proposed model	97.18	97.17	96.71	96.72	96.28	96.27

#### Performance comparison of the models with different numbers of channels

The proposed model uses a three-channel structure with different filter sizes. To investigate the effect of models with different numbers of channels on the experiment, the proposed model is compared with three different number of channels models, such as the one-channel model with filter size 3, the two-channel model with filter sizes 3 and 5, and the four-channel model with filter sizes 3, 5, 7 and 9. The four models all use the same network structure, except for the different number of channels, and are trained with the same hyperparameters. Table 11 shows the performance comparison of the one-channel, two-channel, and four-channel models with the proposed model. From Table 11, it can be found that the number of parameters increases as the number of channels increases, and the proposed model obtains better recognition accuracy.

**Table 11:** Performance comparison of the models with different number of channels.

Models	WISDM			UCI-HAR			PAMAP2		
	Accuracy (%)	F1-score (%)	Parameters	Accuracy (%)	F1-score (%)	Parameters	Accuracy (%)	F1-score (%)	Parameters
One-channel model	93.34	93.53	208,719	94.74	94.68	209,121	94.20	94.18	212,647
Two-channel model	95.23	95.19	416,286	95.86	95.87	417,102	94.78	94.74	423,595
Three-channel model (proposed)	97.18	97.17	623,987	96.71	96.72	625,229	96.28	96.27	634,775
Four-channel model	95.39	95.39	831,822	95.22	95.23	833,502	95.97	95.96	846,187

#### Performance comparison of the proposed model with hybrid models with other RNN variants

The proposed model is a hybrid model combining CNN and GRU, which is a variant of RNN. To discuss the impact of other RNN variants on the experiment, the proposed model is also compared with the CNN-LSTM model and CNN-BiLSTM model. The multichannel architectures of the CNN-LSTM and CNN-BiLSTM hybrid models are the same as that of the proposed multichannel CNN-GRU model except that the LSTM and BiLSTM layers are, respectively, used in the place of GRU layers. Likewise in the case of identical training environments, the experimental results are shown in Table 12. It is obvious that the proposed model achieves higher accuracy with fewer parameters.

**Table 12:** Performance comparison of the proposed model with hybrid models with other RNN variants.

Models	WISDM			UCI-HAR			PAMAP2		
	Accuracy (%)	F1-score (%)	Parameters	Accuracy (%)	F1-score (%)	Parameters	Accuracy (%)	F1-score (%)	Parameters
CNN-LSTM	95.29	95.30	819,059	96.06	96.07	820,301	94.44	94.30	829,847
CNN-BiLSTM	95.77	95.75	2,003,059	96.30	96.28	2,004,301	95.50	95.46	2,014,487
CNN-GRU (proposed)	97.18	97.17	623,987	96.71	96.72	625,229	96.28	96.27	634,775

#### Statistical analysis

To further verify a significant difference between the average performance of each test model, this study uses the non-parametric ranking-based Friedman test for verification. Friedman test firstly gives the corresponding ranking according to the performance of each method on each benchmark data set. In contrast to the references^41^ and^46^, which only provide test results of the iSPLInception and MLSTM-FCN on the UCI-HAR dataset, this work applies the iSPLInception and MLSTM-FCN above to the WISDM and PAMAP2 datasets in the same experimental setting. The accuracy of the iSPLInception on the WISDM and PAMAP2 datasets is 94.84% and 94.93%, respectively, whereas the accuracy of MLSTM-FCN on the WISDM and PAMAP2 datasets is 83.88% and 95.27%. According to the performance of the above test models, the ranking of each test model on the three datasets is shown in Table 13, and then the corresponding values are calculated based on the ranking. The statistic produced in this study is 22.92523, and the p-value is 0.01811. The p-value is less than 0.05, indicating that there is a significant difference between the measurement results of test models.

**Table 13:** Performance ranking of each test model on all benchmark datasets.

Model	Proposed	With classical convolution	One channel-3	One channel-5	One channel-7	Three channels-3,7,11	Two channels	Four channels	CNN-LSTM	CNN-BiLSTM	iSPLInception	MLSTM-FCN
WISDM	1	2	11	10	9	3.5	7	5	6	3.5	8	12
UCI-HAR	1.5	5	12	11	8	6	7	9	4	3	10	1.5
PAMAP2	1	3	12	9	7	6	10	2	11	4	8	5

## Conclusion

In this study, we propose a multi-scale feature extraction fusion model for HAR, which combines the advantages of CNN and GRU to extract local spatial features as well as long-term dependencies in time series data, realizing the automatic extraction of spatio-temporal features from the original data. Meanwhile, three channels with different sizes of convolution kernels are designed for richer features by capturing local dependencies of different limited ranges. In addition, the lightweight recognition accuracy of the sensor-based HAR model is satisfied by carefully designing the architecture of the model and adopting separable convolutions instead of classical convolutions.

We conducted experiments on three benchmark datasets of WISDM, UCI-HAR, and PAMAP2. The results indicate that our method achieves higher recognition accuracy with fewer parameters compared to some existing HAR methods. However, it can be seen from the CM that the recognition accuracy of some activities still needs to be improved. This requires the designed network architecture to extract more accurate and salient features to adapt to different types of activities. Adjusting the DL architecture such as adding the attention mechanism is the focus of subsequent research, making the model run more robustly. Besides, a shallower model is adopted to maintain lower parameters and achieve higher efficiency, but the depth of the model is worth considering later.

## Data Availability

The datasets generated during and/or analyzed during the current study are available at https://www.cis.fordham.edu/wisdm/dataset.php, http://archive.ics.uci.edu/ml/datasets/Human+Activity+Recognition+Using+Smartphones and https://archive.ics.uci.edu/ml/datasets/pamap2+physical+activity+monitoring.
